# Fight or Flight? Alternative Defense of the Pea Aphids, *Acyrthosiphon pisum* on Different Host Plants

**DOI:** 10.3390/insects12070614

**Published:** 2021-07-06

**Authors:** Martin John Martin, Yueming Li, Li Ma, Yi Feng, Zhiqiang Lu

**Affiliations:** 1 Department of Entomology, College of Plant Protection, Northwest A&F University, Yangling 712100, China; martin.john@sua.ac.tz (M.J.M.); 2020050147@nwafu.edu.cn (Y.L.); mlaxt56@nwafu.edu.cn (L.M.); yifeng@nwafu.edu.cn (Y.F.); 2 State Key Laboratory of Crop Stress Biology for Arid Areas, Northwest A&F University, Yangling 712100, China

**Keywords:** pea aphids, host plant, infection, fecundity compensation, winged offspring

## Abstract

**Simple Summary:**

In the event of a pathogen attack, fecundity compensation and production of winged offspring are critical in pea aphids. However, little is known about the effects of the host plant on these responses. The purpose of this study was to investigate the effects of host plant on these two alternative defenses in pea aphids. We took a single adult female from a pink colony of pea aphids that was originally from broad beans and allowed her to reproduce offspring in the laboratory. Some offspring were fed broad beans, while others were fed alfalfa for over 30 generations. We first investigated the backgrounds of their facultative symbionts before infecting them with pathogens and found that the composition of secondary symbionts in our aphid colony was not affected by the host plants. Broad bean reared pea aphids produced more offspring in infected and uninfected conditions, whereas alfalfa reared pea aphids produced more winged offspring when confronting challenges caused by *Staphylococcus aureus* and *Beauveria bassiana*. Our findings showed that the host plant influences the pea aphid’s alternative responses to mortality risks.

**Abstract:**

Non-immunological responses are important alternative strategies for animals to deal with pathogens. It has long been recognized that fecundity compensation and production of winged offspring are two common non-immunological responses used by aphids when confronted with predators or pathogens. However, the effects of host plant on these responses have received little attention. This study investigated the effects of host plant on non-immunological defense in the pea aphids, *Acyrthosiphon pisum,* after bacterial and fungal infections. The aphids were raised in two groups, with one group being raised on broad beans and the other group being raised on alfalfa. The secondary symbiont background was examined, and the aphids were then infected with bacteria and fungus to assess fecundity and winged offspring production. We found that aphids that had been fed alfalfa had fewer offspring than those fed broad beans. Alfalfa-fed aphids produced more winged offspring in response to *S. aureus* and *B. bassiana* infections. Our findings suggest that the host plant plays a key role in fecundity and winged offspring production in pea aphid colony.

## 1. Introduction

When encountering invading pathogens, insects mount immunological responses to defend themselves. These responses include both instant responses, such as phenoloxidase-mediated melanization [1] and the burst of reactive oxygen species [2], which typically occur within minutes to hours of pathogen recognition as non-self, and inducible responses, such as NF*κ*B-mediated antimicrobial peptide synthesis [3,4]. These effector and intermediate molecules produced by these responses either directly kill pathogens or limit their spread in host insects.

Besides immune responses, insects may employ alternative, non-immunological defenses to cope with pathogens. These non-immunological defense strategies include behavioral avoidance, hygienic behavior in social insects, behavioral thermoregulation, self-medication, reduced feeding, fecundity compensation, and so on (see the review by Parker et al. [5]). Non-immunological defenses can be critical for insects to survive pathogens and parasites, particularly for those that have an incomplete and limited immune system, such as the pea aphid *Acyrthosiphon pisum* [6]. Pea aphids can detect the bacterium *Pseudomonas syringae*, which produces the fluorescent compound pyoverdine, and avoid feeding on leaves contaminated by this pathogen [7]. An early study suggested that pea aphids may increase reproduction in response to death threats [8]. It has later found that bacterial infection resulted in increased reproduction in pea aphids [9,10]. Insects may attempt to flee when they recognize a threat to their survival. In the presence of predatory ladybirds, pea aphids will produce more winged morphs [11]. When infected with fungal pathogens but not with bacterial pathogens, pea aphids produce more winged morphs than uninfected aphids [12,13]. Winged aphids can escape infected patches, though they are less resistant to pathogens than wingless morphs [14]. 

Aphid fecundity and wing dimorphism are profoundly affected by a variety of factors, especially by population density and host plant species and quality [15,16,17,18]. A recent study demonstrated that the presence of ladybird predators induced the production of more winged morphs in the pea aphids specialized on alfalfa, but this was not observed in the lineage of those specialized on clover, suggesting that the influence of host plant specialization on wing dimorphism phenotypic responses [19]. We speculate that insects may employ different alternative defensing strategies in different ecological conditions in response to infections.

Despite the fact that fecundity compensation and winged offspring production are two common alternative defense responses of pea aphids, the role of host plants in these responses remains largely unexplored, and it is also unknown whether bacteria can induce winged offspring production in wingless adult pea aphids. Therefore, in this study, we investigated the effects of host plants on these two alternative pea aphid defense responses as well as investigating whether bacteria could induce winged offspring production in these insects. We believe that a thorough investigation on the roles of host plants and pathogens in alternative pea aphid defenses will provide useful information for integrated pest management programs for these insects.

## 2. Materials and Methods

### 2.1. Pea Aphids Rearing

We used a pink colony of pea aphids collected from broad beans in Gansu, China in the spring of 2018. We chose one adult wingless female from the colony and allowed her to reproduce on broad beans in an incubator set to a temperature of 20 ± 1 °C, a photoperiod of 16L:8D, and a relative humidity of 70 ± 5%. Some of her offspring then continued to feed on broad beans (*Vicia faba*) while some were shifted to alfalfa (*Medicago sativa*) and reared for more than 30 generations. At the end of each generation, ten adult aphids (10–12 days old) were placed on their respective host plants for 24 h. The plants hosting these aphids were housed in a plastic box covered with a muslin cloth on the top for ventilation. The adults were removed the following day, leaving the offspring to feed on the plants and grow to adulthood. The aphids were all kept in the incubator with the settings described above. In the following experiments, newly matured wingless adult aphids were used.

### 2.2. Infection of Pea Aphids with Bacteria Staphylococcus aureus and Escherichia coli

Bacteria were prepared, and the pea aphids were infected as previously described [20]. Briefly, the bacteria were cultured to logarithmic phase at an optical density (OD_600nm_) of about 1 and harvested by means of centrifugation at 5000 rpm for 10 min. The bacterial cells were re-suspended in sterile saline (0.85% NaCl) and adjusted to a concentration of about 2 × 10^11^ and 1 × 10^10^ for *S. aureus* (ATCC43300) and *E. coli* (DH5α), respectively. Aphids were placed in petri dishes and kept on ice to anaesthetize them. A sterile capillary tube with a long tip (about 4 mm) was dipped in the bacteria suspension before being pricked dorsolaterally through the abdominal wall into the aphid at a depth of about 1 mm. Aphids in the control group were pricked with sterile saline solution. Each group contained ten adult aphids. After treatment, the aphids were returned to their host plants. For the next three days, fecundity was recorded on a daily basis [21]. Offspring born on the first and second days were counted and removed from the plants to avoid overcrowding, while offspring born on the third day were counted and transferred to three host plants, with each plant hosting ten offspring. These offspring were raised to the fourth instar, where winged offspring could be examined [21]. The infection and winged/wingless examination were carried out independently three times.

### 2.3. Infection of Pea Aphids with Fungus Beauveria bassiana

Fungus *Beauveria bassiana* (Strain 242) was grown on potato dextrose agar (PDA) at 28 °C until the media was full of white hyphae and conidia [20]. The spores were collected by flooding the media with sterile 0.85% sodium chloride containing 0.05% Tween-20 and were then pipetted into a 1.5 mL Eppendorf tube, mixed thoroughly, transferred to another tube (1.5 mL Eppendorf), and enclosed in a 0.5 mL Eppendorf tube filled with absorbent cotton wool. To separate spores from hyphae, the mixture was centrifuged at 8000× *g* for 5 min. The spores were counted using an improved Neubauer chamber (Lauda-Königshofen, Germany) under an inverted research microscope (ECLIPSE TE2000-S, Nikon, Tokyo, Japan). Final concentration (6.1 × 10^8^ spores/mL) was adjusted using sterilized distilled water. Infection of the aphids was performed as described by Hatano et al. [12] with some modifications, specifically in that the infection arena was not made up of leaves wrapped in filter paper on the petioles, but rather was a petri dish with filter paper at the base and a lid. The spore suspension was then smeared on the filter paper at the base and lid of a petri dish. The infection arena was left to dry, after which fifteen adult aphids were placed into the arena and returned to the incubator for 24 h. After that, the aphids were transferred to their respective host plants, and fecundity was recorded every day for three days [21]. Offspring born on the first and second days were counted and discarded, whereas those born on the third day were counted and transferred to three plants, each of which hosted ten offspring. Aphids in the control group were exposed to the same arena but with 0.85% sodium chloride containing 0.05% Tween-20. At the fourth instar, both winged and wingless individuals were examined. Infection and examination of wing presence/absence was performed three times independently.

### 2.4. Statistical Analysis

All analyses were carried out with R software version 4.0.3 (R Development Core Team, 2020). Data were modelled using generalized linear models (GLM) without transformation [22]. Host plants (Broad bean and alfalfa) and treatments (Saline, *E. coli*, *S. aureus*, and *B. bassiana*) were treated as fixed effects. Fecundity was analysed with negative binomial distribution. The data regarding the proportion of winged offspring was considered as zero inflated [23,24], therefore, the zero-inflated negative binomial was used with the zeroinfl function in the pscl package [25]. For all models, the ANOVA function in the car package [26] was used for the analysis of deviance, the Nagelkerke function was used for the determination of *p*-values, and the pseudo-*R*-squared value was used for each model. Post hoc analysis was conducted with the emmeans package and the pairwise comparison was done using the Sidak method.

## 3. Results

### 3.1. Host Plants Affect the Fecundity of the Pea Aphids in Both Infected and Uninfected Conditions 

**Figure 1:**
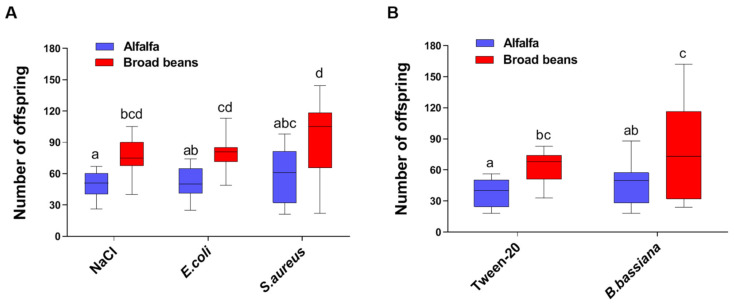
Effect of bacterial and fungal infections on the fecundity of adult pea aphids feeding on different host plants. Shown are the total number of offspring produced in three consecutive days after challenging the adult aphids with bacteria (**A**) and fungus (**B**) (*n =* 9). The box and whiskers plots indicate the minimum to a maximum number of offspring. The horizontal lines indicate the mean values, and the vertical bars indicate the SE of the repeats. Different letters between groups indicate *p* < 0.05.

In the bacteria infection experiments, we found that host plants have significant effect on fecundity (*χ*^2^ = 25.67, df = 1, *p* < 0.0001), whereas the treatments had no significant effect (*χ*^2^ = 1.64, df = 2, *p* = 0.20). Likewise, the interaction between host plants and treatments showed no influence on fecundity (*χ*^2^ = 0.07, df = 2, *p* = 0.93). A pairwise comparison revealed that the aphids reared on alfalfa and injected with sterile saline produced fewer offspring than those reared on broad beans and treated the same way (*p* < 0.0001). Similarly, aphids reared on alfalfa and infected with *E. coli* or *S. aureus* produced fewer offspring than their broad bean counterparts infected with the same pathogens (Figure 1A, *p* < 0.0001). On the other hand, in the experiments where the aphids were infected with fungi (*B. bassiana*, Figure 1B), the host plants had significant effect on the fecundity (*χ*^2^ = 11.70, df = 1, *p* = 0.0017) but not the treatments (*χ*^2^ = 2.01, df = 1, *p* = 0.17). There was no interaction between effects of host plants and treatments on the fecundity (*χ*^2^ = 0.03, df = 1, *p* = 0.87). A multiple comparison analysis revealed that aphids reared on alfalfa and exposed to 0.05% Tween-20 produced fewer offspring than their counterparts reared on broad beans (*p* = 0.0028). In the infected condition, aphids reared on alfalfa produced significantly fewer offspring than those reared on broad beans (*p* = 0.0028). Taken together, our findings suggest that host plants play a key role in the fecundity of pea aphids in both infected and uninfected conditions.

### 3.2. Pea Aphids Produce Winged Offspring in Response to Pathogens Differently between Plants

**Figure 2:**
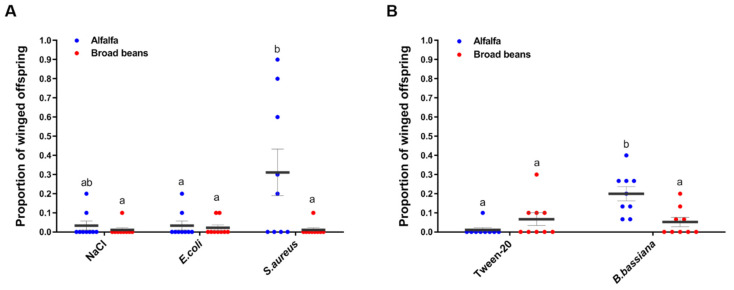
The proportion of winged offspring produced by adult pea aphids after bacterial and fungal infections. The adult aphids were challenged with bacteria (**A**) and fungal spores (**B**) (*n* = 9). Each dot represents winged offspring from an individual aphid. The bars show the mean values ± SEM. Different letters indicate *p* < 0.05.

In the bacterial infection experiment, we found that the host plant from which pea aphids feed had a significant impact on the production of winged offspring (*χ*^2^ = 21.34, df = 1, *p* < 0.0001, Figure 2A). The treatments with bacteria had a significant effect on production of winged offspring (*χ*^2^ = 80.65, df = 2, *p* < 0.0001) as well. Furthermore, the interaction between the treatments and the host plants significantly affected the production of winged offspring (*χ*^2^ = 9.28, df = 2, *p* = 0.01). In the infected condition, adult aphids raised on alfalfa and subjected to *S. aureus* infection produced more winged offspring than broad their bean-reared counterparts (Figure 2A, *p* = 0.02). On the contrary, the number of winged offspring produced after infecting the aphids with *E. coli* did not differ much between aphids reared on either plant (*p* > 0.05). In the infected condition, adult aphids raised on alfalfa and subjected to *S. aureus* infection produced more winged offspring than their broad bean-reared counterparts (Figure 2A, *p* = 0.02). On the contrary, the number of winged offspring produced after infecting the aphids with *E. coli* did not differ much between aphids reared on either plant (*p* > 0.05). In the fungal infection experiment, the model showed that the host plants had significant effects on the production of winged offspring by adult wingless aphids (*χ*^2^ = 6.19, df = 1, *p* = 0.01, Figure 2B). In contrast, the model did not detect any significant influence of the treatments on the number of winged offspring produced (*χ*^2^ = 0.12, df = 1, *p* = 0.73). However, the interaction between the host plants and the treatments was statistically significant (*χ*^2^ = 6.09, df = 1, *p* = 0.01). As above, we conducted a pairwise comparison between the treatments and found that in the infected condition, aphids reared on alfalfa produced more winged offspring than those reared on broad beans (*p* < 0.0001). In the uninfected condition (Tween-20), we did not detect significant difference between aphids reared on alfalfa or on broad beans (*p* = 0.21). More winged offspring were produced by aphids reared on alfalfa after being challenged by *B. bassiana* (*p* < 0.0001, Figure 2B). These data demonstrate that Gram-positive bacterium *S. aureus* and fungus *B. bassiana* infections significantly induced winged offspring production, and the host plants influence the responses of the aphids to infections.

## 4. Discussion

Non-immunological responses as alternative defense strategies in pea aphids have not been thoroughly studied. In the current study, a pea aphid colony was collected from broad beans, and part of it was reared on alfalfa while the other part was reared on broad beans. In the diagnosis of secondary symbionts (Supplementary Material, the primers used for diagnosis PCR are listed in Table S1 [27,28,29,30]), only *Serratia symbiotica* was found in both groups of aphids (Figure S1), suggesting that transferring aphids to alfalfa had no effect on the composition of the facultative symbionts. Aphid colonies with different secondary symbionts have been shown to influence the aphid response to stress [12,21]. Our results showed that in both infected and uninfected conditions, pea aphids reared on broad beans produced more offspring than those reared on alfalfa (Figure 1). Furthermore, the infection of pea aphids with Gram-positive bacterium *S. aureus* and fungus *B. bassiana* induced the production of winged offspring in the aphids that were fed alfalfa (Figure 2).

Previous research revealed that pea aphids could invest in terminal reproduction in response to microbial threats [8]. Infection with Gram-negative bacteria Enterobacter Ng5b and *Pseudomonas syringae* resulted in increased fecundity in pea aphids [9,10]. On the contrary, exposure to fungi *B. bassiana*, *Pandora neoaphidis*, and *Zoophthora occidentalis* reduce fertility [9,31]. We found that infection of the aphids with bacteria or fungus had no significant impact, but the host plant on which the aphids were reared had a significant impact on the number of progeny they produced. Aphid reared on broad beans produced more offspring in both infected and uninfected conditions (Figure 1). It was proposed that the presence of different symbiotic bacteria modulates aphid responses to pathogens [9]. Therefore, the discrepancies between these studies could be attributed to the different symbiotic background of the aphids [12,21].

We also found that the aphids that were fed alfalfa produced a higher proportion of winged offspring when they were challenged by *S. aureus* and fungus *B. bassiana* but not with *E. coli* or sterile stab (control). This may imply that Gram-positive bacteria and fungi both stimulate the production of winged offspring in pea aphids, but this is dependent on the host plant that the insects feed on. The winged aphids are able to fly away from pathogens, though they possess weaker immune response than wingless aphids [14]. Early studies showed that the presence of predators or the challenge of fungi resulted in more winged offspring produced by pea aphids [11,12]. However, the production of winged offspring did not increase when challenged by *E. coli* [13]. In a recent study, the induction of winged offspring by predators was observed in alfalfa biotype aphids, but was not significant in the clover biotype, indicating the impact of host plant on wing dimorphism responses [19]. Aphids harboring different symbiotic bacterium *Regiella* strains showed variation in the proportion of winged offspring induced by crowding stress [21]. Winged phenotype induction by means of fungal infection was significantly affected by dosage and aphid line with different symbiotic bacteria [32]. It is not surprising, then, that there is inconsistency in the literature on winged offspring production induced by infection given that different aphid lines, host plants, and pathogens were used in different studies. This could also imply that the morphological alterations caused by infection is the result of complex and intricate interactions between aphids, pathogens, host plants, and symbionts.

The most intriguing finding in our study is that the aphids produced more winged offspring on alfalfa, but not on broad beans, in response to *S. aureus* and *B. bassiana* infections. The aphids fed on broad beans produced more offspring than those fed on alfalfa in both infected and uninfected conditions, implying that broad beans may provide aphids with higher quality nutrition than alfalfa. Therefore, when the food quality (or maybe availability) is low, the aphids will produce winged offspring that are able to fly away and search for new food sources. It has been noticed for a long time that host plant quality affects the production of winged versus wingless morphs in aphids [15,16,18]. Studies in aphids and brown plant hoppers have revealed that carbohydrate level and the insulin signaling pathway regulate long wing/short wing and winged/wingless dimorphism [33,34]. It was discovered that red pea aphids reared on low-quality plants turned pale, had lower lipid and carbohydrate contents, and a faster dispersal speed. This may demonstrate the significance of nutrition in the morphological alterations that are induced in response to deprived food sources [35].

Our findings therefore show that host plants have a major impact on terminal reproduction and winged offspring production in pea aphids as a response to the risk of mortality. How the host plant regulates the switch between terminal reproduction and the induction of winged offspring remains to be elucidated.

## 5. Conclusions

Aphids that were fed alfalfa had less offspring overall but had more winged offspring when exposed to Gram-positive bacterium *S. aureus* and fungus *B. bassiana,* whereas aphids fed broad beans had a higher number of offspring but had less winged offspring after being infected with pathogenic bacteria and fungus. 

## Supplementary Materials

The following are available online at https://www.mdpi.com/article/10.3390/insects12070614/s1, Figure S1: Diagnosis of eight known secondary symbionts in the pea aphids feeding on alfalfa and on broad beans, Table S1: Primer sequences used for detection of secondary symbionts in the pea aphid.
