# Supplementary material for: Fight or Flight? Alternative Defense of the Pea Aphids, Acyrthosiphon pisum on Different Host Plants
Source: Insects. 2021 Jul 6;12(7):614. doi: 10.3390/insects12070614 (PMC8306235; doi:10.3390/insects12070614)
Supplement: Supplementary file 1 [file insects-12-00614-s001.zip › insects-1241947-supplementary.pdf]

insects

MDPI

Supplementary Material

Diagnosis of secondary symbionts in the pea aphids

We used PCR to detect the presence of eight known symbionts in the aphids after over 30 generations on alfalfa and broad beans, respectively, to see if rearing on different host plants changes the secondary symbiotic bacteria in our pea aphid colony. Five adult pea aphids were used for DNA extraction with a TIANamp Stool DNA Kit (Tiangen Biotech, China). PCR detection of eight secondary symbionts (*Hamiltonella defensa*, *Regiella insecticola*, *Serratia symbiotica*, *PAXS*, *Rickettsia sp.*, *Spiroplasma sp.*, *Rickettsiella sp.*, *and Wolbachia sp.*) was performed using specific primers targeting the symbiotic bacterial genes (Supplemental table 1). The total PCR reaction volume was 50 μl including 0.25 μl TaKaRa Taq (R001A, Takara Bio Inc., China), 5 μl 10X PCR Buffer (with Mg^{2+}), 4 μl dNTP mixture (2.5 mM), 1.75 μl DNA template, 1μl forward and reverse primers each, and 37 μl ddH_2O. The PCR reactions were performed on a MiniAmp™ Plus Thermal Cycler (A37835, Thermal Fisher Scientific) with the following condition: 94℃ for 3 min, 35 cycles consisting of 94℃ for 30 sec, 52℃ for 45 sec and 72℃ for 1 min. Four microlitres of PCR reaction was electrophoresed on 1% agarose gel and visualized under a UV detector. Only *Serratia symbiotica* was found in these two groups fed alfalfa and broad beans (Supplemental figure 1), indicating that the host plants had no effect on the composition of secondary symbionts in the pea aphid colony used in this study.

Table S1. Primer sequences used for detection of secondary symbionts in the pea aphid.

Symbionts	Primer sequences (5′→ 3′)	References
Hamiltonella defensa	Forward: 10F<br>AGTTTGATCATGGCTCAGATTG<br>Reverse: T419R<br>AAATGGTATTCGCATTTATCG	[27]
Regiella insecticola	Forward: 10F<br>Reverse: U443R<br>GGTAACGTCAATCGATAAGCA	[27]
Serratia symbiotica	Forward: 10F<br>Reverse: R443R<br>CTTCTGCGAGTAACGTCAATG	[27]
PAXS	Forward: 10F<br>Reverse: GCAACACTCTTTGCATTGCT	[27]
Rickettsia	Forward: 16SA1 AGAGTTTGATCMTGGCTCAG<br>Reverse: TTTGAAAGCAATTCCGAGGT	[28]
Spiroplasma	Forward: 16SA1<br>Reverse: ATCATCAACCCTGCCTTTGG	[28]
Rickettsiella	Forward: GGGCCTTGCGCTCTAGGT<br>Reverse: TGGGTACCGTCACAGTAATCG	[29]
Wolbachia	Forward: GGGTCCAATAAGTGATGAAGAAAC<br>Reverse: TTAAAACGCTACTCCAGCTTCTGC	[30]

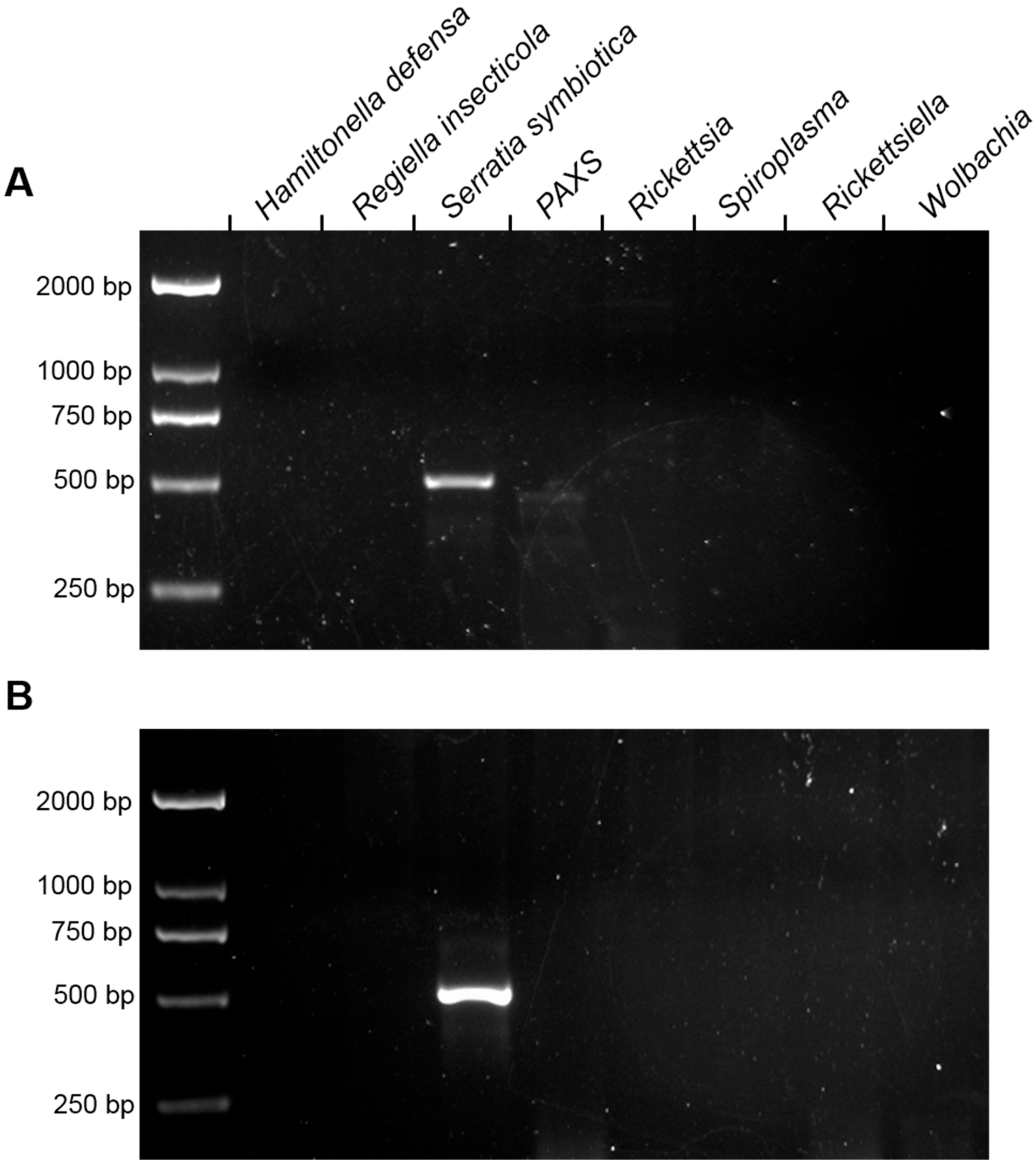

Figure S1. Diagnosis of eight known secondary symbionts in the pea aphids feeding on alfalfa (A) and on broad beans (B). DNA from five aphids on alfalfa or broad beans was prepared and used as the templates for diagnosis PCR. The reactions were separated on 1% agarose gels and photographed under a UVP image system.

References

27. Ferrari, J.; West, J.A.; Via, S.; Godfray, H.C. Population genetic structure and secondary symbionts in host associated populations of the pea aphid complex. *Evolution* **2012**, *66*, 375-390.

28. Fukatsu, T.; Nikoh, N. Two intracellular symbiotic bacteria from the mulberry psyllid *Anomoneura mori* (Insecta, Homoptera). *Appl. Environ. Microbiol.* **1998**, *64*, 3599-3606.

29. Tsuchida, T.; Koga, R.; Horikawa, M.; Tsunoda, T.; Maoka, T.; Matsumoto, S.; Simon, J.-C.; Fukatsu, T. Symbiotic Bacterium Modifies Aphid Body Color. *Science* **2010**, *330*, 1102.

30. Tsuchida, T.; Koga, R.; Shibao, H.; Matsumoto, T.; Fukatsu, T. Diversity and geographic distribution of secondary endosymbiotic bacteria in natural populations of the pea aphid, *Acyrthosiphon pisum*. *Mol. Ecol.* **2002**, *11*, 2123-2135.
